# Sleep Quality and Associated Factors Among Medical Students in Tropical China: A Cross-Sectional Study in Hainan Province

**DOI:** 10.3390/healthcare14070908

**Published:** 2026-04-01

**Authors:** Li-Qin Fu, Zhao-Xin Wang, Xin-Yi Li, Di-Er Cheng, Zhen Zhou, Hou-Qian Shan

**Affiliations:** 1 School of Public Health, Hainan Medical University, Haikou 571199, China; fuliqin63@muhn.edu.cn (L.-Q.F.);; 2 School of Pharmacy, Hainan Medical University, Haikou 571199, China; 3 First Clinical Medical School, Hainan Medical University, Haikou 571199, China; 4 UWE College, Hainan Medical University, Haikou 571199, China

**Keywords:** academic stress, influencing factors, medical students, sleep quality, tropical region

## Abstract

**Background:** Sleep problems are prevalent among student populations worldwide. Medical students, facing heavy academic workloads and intense pressure, are particularly susceptible to sleep disorders. While sleep quality among Chinese university students has been consistently declining, research focusing on medical students in tropical island provinces like Hainan remains insufficient. This study aims to address this geographical gap by analyzing the sleep quality status and influencing factors among medical students in Hainan Province. **Objective:** To investigate the current status of sleep quality and its associated factors among medical students in Hainan Province, providing a scientific basis for developing targeted interventions. **Methods:** A cross-sectional survey was conducted in April 2024 using purposive sampling to recruit undergraduate students from a medical university in Hainan. The Self-Rating Scale of Sleep (SRSS) developed by Li Jianming was administered, and 551 valid questionnaires were collected anonymously. Data were analyzed using univariate analysis and pairwise comparisons to assess sleep quality and associated factors, with demographic variables as independent variables. **Results:** Among participants, 40.1% reported sleep problems (31.2% mild, 8.2% moderate, 0.7% severe). The mean total SRSS score was 21.78 ± 5.73. Compared to the national norm, medical students showed significantly higher scores in sleep quality, insufficient arousal, and post-insomnia responses (*p* < 0.05). Academic major was identified as a significant influencing factor (*p* = 0.012), with clinical medicine students demonstrating significantly poorer sleep quality than health management majors (*p* = 0.010). No significant differences were found for gender or academic year. **Conclusions:** Sleep problems are prominent among medical students in Hainan, with clinical medicine students at higher risk due to academic and professional pressures. Recommendations include optimizing curriculum schedules, strengthening psychological support systems, and developing targeted interventions for clinical majors.

## 1. Introduction

Sleep is a natural, cyclical physiological state. It is fundamentally important for humans, serving not only to alleviate physical fatigue but also to rapidly restore mental alertness, thereby constituting an essential component of overall well-being [1]. Furthermore, sleep regulates bodily functions and moderates metabolic processes [2]. The importance of sleep has been underscored in the “Sleep and Health” report jointly published by the World Health Organization (WHO) and the World Federation of Sleep Research Societies (WFSRS), which emphasizes that quality sleep plays a critical role in maintaining health [3].

Globally, numerous countries are confronting sleep issues. In the United States, 33.2% of adults report inadequate sleep duration, while in Japan, 14.5% of women and 12.2% of men experience insomnia symptoms [4]. This problem is particularly pronounced among student populations. A Japanese study found that 52.6% of students exhibited insomnia symptoms and 38.2% reported sleeping less than six hours daily [5]. Similarly, approximately 39% of students in Brazil face sleep-related problems, while in the United States, about 17.9% resort to medication to improve their sleep [6]. International research focusing specifically on medical students also reveals a high prevalence of sleep problems. For example, a meta-analysis by Chaabane et al. [7] involving 150 studies across 16 countries in the Middle East and North Africa reported a pooled prevalence of poor sleep quality among medical students of 59.1%. Khaled et al. [8] found that as many as 88.1% of medical students in Saudi Arabia experienced sleep problems, while Vidović et al. [9] reported that 67.9% of medical students in Croatia had poor sleep quality. A study by Bousgheiri et al. [10] in the Mediterranean region further confirmed that sleep problems are common among medical students.

In China, the “2022 China Sleep Quality Report” indicates that in 2021, the average daily sleep duration among the general population was 7.06 h, representing a decrease of 1.5 h compared to 2012 [11]. Following the 2021 implementation of the national “Double Reduction” policy aimed at alleviating academic burdens for compulsory education students, 61.53% of primary and secondary school students now achieve at least 8 h of sleep on weekdays. In contrast, university students face distinct challenges—such as employment pressure and academic planning—that frequently disrupt their sleep and contribute to a higher prevalence of sleep disorders. This population is therefore considered particularly vulnerable to sleep issues, the quality of which significantly impacts their physical health, mental well-being, and overall psychological state. Currently, sleep issues are progressively intensifying. Specifically, 27.52% of university students report “always” going to bed later than intended, while 26.80% indicate this happens “sometimes” [1]. As a distinct subgroup within the broader university student population, medical students face more intense competition, a more complex and demanding curriculum, and a longer period of study. They were selected as the study population for two main reasons: on the one hand, they represent the future backbone of healthcare, and their physical and mental health is directly tied to the quality of future medical services; on the other hand, research by Zhou et al. [12] indicates that medical students sleep less than their non-medical peers, with the prevalence of sleep disorders in this group ranging from 22.40% to 52.84% [13], making their sleep issues more pronounced than those of students in other disciplines. Furthermore, the impact of sleep problems among medical students often extends beyond their time in school.

After graduation, they may face new challenges as they enter clinical practice. The transition from medical school to standardized residency training involves a shift from the classroom to the clinical setting, where they must contend with shift work, night duties, and more complex doctor–patient relationships—factors that can lead to significant changes in sleep patterns [14,15,16]. A systematic review and meta-analysis by Shafiee et al. [14] on medical students and residents in Iran found a high overall prevalence of sleep disorders in this population, with residents facing distinct stressors compared to their time in medical school. A study by Moderie et al. [15] in Canada reported that 51.7% of residents experienced sleep disorders, with an average sleep debt of 1.59 h, and found a significant association between night shift work and poor sleep quality. Once physicians enter the stable phase of their careers, ongoing clinical pressures and the long-term demands of professional development may also result in sleep patterns that differ from those observed during medical school [16]. Similarly, a study by Fabiyi et al. [16] in Nigeria found significant differences in sleep quality across different stages of residency training, suggesting that the transition from student life to professional practice is a critical period for changes in sleep patterns and warrants further attention [14,15,16].

When individuals experience sleep problems, it can precipitate a range of mental health issues, such as depression and anxiety. Statistics indicate that the detection rate of depression among Chinese university students reaches 13.2% to 42.1% [17]. Furthermore, students with sleep problems report a higher incidence of physical ailments and academic difficulties compared to those with sound sleep [18]. Sleep disorders can also diminish physical well-being and impair an individual’s ability to manage family, academic, and social responsibilities, thereby adversely affecting their overall quality of life [19]. According to the “2024 China Sleep Quality Report” [20], the average sleep duration was 5.16 h, and the composite sleep score was 62.61, marking the lowest figures in the past three years and reflecting a decrease of 5.16 points since 2022. More concerning is the growing prominence of bedtime procrastination compared to previous years. These data are derived from a nationwide online survey on residents’ sleep conducted in December 2023 by the Institute of Sociology, Chinese Academy of Social Sciences. The sample covered 27 provincial-level administrative regions (excluding Hong Kong, Macao, Taiwan, Tibet, Qinghai, Hainan, and Ningxia). It is noteworthy that existing national sleep surveys often fail to adequately represent regions with unique geographical and climatic characteristics, such as Hainan—China’s only tropical province.

Therefore, this study focuses on Hainan Province, a tropical region in China, and specifically examines the medical student population to analyze sleep quality and explore influencing factors. To address these concerns, this research utilized the Self-Rating Scale of Sleep (SRSS) developed by Li Jianming to assess the sleep quality of medical students in Hainan. The study aims to provide a scientific basis for improving sleep quality in this population and to offer evidence-based references for medical colleges in developing targeted interventions.

## 2. Methods

### 2.1. Study Participants

The target population consisted of all undergraduate students at a medical university in Hainan, totaling approximately 11,800 individuals. A purposive sampling method was used to select undergraduate students enrolled in medicine-related programs at the university as survey participants. The survey was conducted in April 2024, during a regular teaching period when no major examinations were scheduled at the university. The required sample size was calculated using the formula $n=z^{2}\times p\times \left(1-p\right)÷d^{2}$, with a 95% confidence level (Z = 1.96), an expected prevalence of sleep problems of 50% (*p* = 0.5) to maximize the sample size, and a margin of error of 5% (d = 0.05). The minimum required sample size was estimated to be 385. Accounting for a 10% rate of invalid responses, the final target sample size was set at 424. The electronic questionnaire was distributed via the Questionnaire Star platform, and a total of 551 valid responses were collected, meeting the required sample size.

### 2.2. Instruments

An anonymous questionnaire was administered using the Self-Rating Scale of Sleep (SRSS), developed under the direction of Professor Li Jianming [21]. In this study, the scale demonstrated acceptable internal consistency, with an overall Cronbach’s α of 0.786, which exceeds the standard reliability threshold of 0.700. The instrument consists of 10 items, in addition to basic demographic questions. Each item is rated on a 5-point Likert scale from 1 to 5 (where 1 represents “never” and 5 represents “always” or “severe”). The sum of all item scores yields a total score ranging from 10 to 50. Lower total scores indicate fewer sleep problems, with the minimum score of 10 representing an essentially normal sleep profile. Conversely, higher total scores indicate more severe sleep issues. Based on a standardized assessment of 13,273 healthy individuals, Li Jianming et al. [22] established a national norm for the SRSS. According to this norm, a total score of 22 or below is classified as normal sleep, while a score of 23 or above indicates the presence of a sleep problem. The severity is further stratified as follows: scores of 23–29 signify mild sleep problems, 30–39 moderate sleep problems, and 40–50 severe sleep problems.

### 2.3. Study Variables

The variables in this study comprised the following two categories:

Demographic variables: These included sex, age, ethnicity, place of origin, academic year, major, household registration, birth order, monthly living expenses, grade point average (GPA), student leader status, paternal education level, maternal education level, and family structure. All of these variables were designed by the researchers based on the study objectives and were collected directly from the general information section of the questionnaire.

Sleep quality variables: The total SRSS score was used as a continuous variable to measure sleep quality. In addition, sleep quality was categorized based on the national norm [22] into normal sleep, mild sleep problems, moderate sleep problems, and severe sleep problems. The scores for the ten SRSS subscales (i.e., individual factors) were also included to characterize specific aspects of sleep and to enable comparison with the national norm. All of these variables were calculated by the authors based on the scoring criteria of the SRSS.

### 2.4. Statistical Analysis

A database was established for statistical analysis. Continuous data are presented as the mean ± standard deviation ($\bar{x}\pm s$). Prior to analysis, the normality of continuous variables was assessed using the Kolmogorov–Smirnov test.

For the univariate analysis, the SRSS total score was treated as the dependent variable, while students’ general demographic characteristics served as independent variables. The specific analytical methods employed were as follows: independent samples t-test for dichotomous variables, one-way analysis of variance (ANOVA) with post hoc comparisons using Bonferroni correction for multi-categorical variables, and Pearson’s correlation analysis for continuous variables. In cases where the assumption of homogeneity of variance was violated for the ANOVA, the non-parametric Kruskal–Wallis H test was applied for multi-categorical comparisons, and Welch’s ANOVA was considered as an alternative robust test. The threshold for statistical significance was set at α = 0.05.

### 2.5. Ethical Considerations

This study was conducted in accordance with the Declaration of Helsinki and approved by the Institutional Review Board of Hainan Medical University. The digital questionnaire was distributed via online platforms, and all participants provided informed consent before participation. Anonymity and data confidentiality were strictly maintained throughout the study. Detailed information regarding ethical approval (protocol code HYLL-2024-587), informed consent, and data availability is provided in the Declarations Section at the end of this manuscript.

## 3. Results

### 3.1. Demographic Characteristics of the Study Participants

The target population of this study comprised approximately 11,800 undergraduate students at Hainan Medical University. The sample size of 551 exceeded the minimum required sample size of 385 (calculated with a 95% confidence level and 5% margin of error), ensuring adequate statistical power for the analyses. The sample included students from all academic years (2020–2023 cohorts) and covered all major medical disciplines offered at the university, suggesting that the sample is broadly representative of the target population.

Among the 551 students surveyed, 197 (35.8%) were male and 354 (64.2%) were female. In terms of grade, sophomores constituted the largest group (346, 62.8%), followed by freshmen (18.5%), seniors (13.8%), and juniors (4.9%). Regarding academic majors, the distribution was as follows: Clinical Medicine (139, 25.2%), Preventive Medicine (58, 10.5%), Health Management (285, 51.7%), Medical Laboratory Science (59, 10.7%), and other majors (10, 1.8%). The Han ethnicity was predominant (457, 82.9%), with Li ethnicity (50, 9.1%) and other minority groups (44, 8.0%) comprising smaller proportions. The distribution of students by geographical origin and household registration was balanced: 51.2% hailed from rural areas and 48.8% from towns/cities, while 44.1% were local Hainan students and 55.9% were from other provinces. In terms of family structure, nuclear families were most common (426, 77.3%), followed by single-parent families (46, 8.3%) and stem families (43, 7.8%). The educational attainment of the parents was generally low. Specifically, 58.6% of fathers and 65.3% of mothers had a junior high school education or below. For fathers, 20.0% had completed high school, and 21.4% had attained a college degree or higher; the corresponding figures for mothers were 16.7% and 18.0%, respectively. Additionally, 34.3% of the students held student leadership positions. Grade Point Averages (GPAs) were concentrated in the medium to high ranges, with 38.3% of students having a GPA between 3.0 and 3.49, and 34.1% having a GPA of 3.5 or higher. See Table 1 for details.

### 3.2. Overall Sleep Quality of Medical Students

The mean total SRSS score for the 551 medical students was 21.78 ± 5.73. Among the participants, 330 students (59.89%) were classified as having no sleep disorder, comprising 124 males and 206 females. Conversely, 221 students (40.1%) were identified as having some form of sleep problem. Of these, 172 (31.2%) had mild sleep problems, 45 (8.2%) had moderate sleep problems, and 4 (0.7%) had severe sleep problems.

### 3.3. Comparison of SRSS Factor Scores Between Medical Students and the National Norm

The survey results indicated that scores for the factors of Sleep Quality, Insufficient Arousal, Difficulty Falling Asleep, Sleep Instability, Early Awakening, Nightmare-induced Awakening, and Post-insomnia Response were statistically significantly different from the national norm (*p* < 0.05). Specifically, the medical students scored significantly higher than the norm on Sleep Quality, Insufficient Arousal, and Post-insomnia Response. This pattern suggests that they experienced subjectively poorer sleep quality, lower daytime alertness, and more pronounced physical and mental reactions following insomnia compared to the general population. Conversely, their scores for Early Awakening, Sleep Instability, and Nightmare-induced Awakening were significantly lower than the norm, indicating better sleep continuity and a lower frequency of early morning awakenings and nightmares. No statistically significant differences were observed for the factors of Sleep Insufficiency, Sleep Duration, Medication Use, or the Total SRSS Score (*p* > 0.05). This indicates that the medical students were generally comparable to the national population in terms of total sleep time, use of sleep medication, and the overall level of sleep disturbance. See Table 2 for details.

### 3.4. Univariate Analysis Results

This study employed univariate analysis to examine the influence of various factors on SRSS scores within the medical student cohort. The results revealed that academic major had a statistically significant influence on SRSS scores (*p* < 0.05). In contrast, other factors—including gender, age, ethnicity, geographical origin, grade, household registration, birth order, monthly allowance, grade point average (GPA), student leadership role, paternal education level, maternal education level, and family structure—demonstrated no statistically significant association with SRSS scores (*p* > 0.05).

These findings indicate that academic major may be an important factor influencing sleep quality in this population, whereas the other demographic and sociological factors examined in this study did not demonstrate a significant influence. See Table 3 for details.

### 3.5. Pairwise Comparisons of Sleep Scores Across Different Majors

To further investigate the differences in sleep scores among the various academic majors, pairwise comparisons were conducted, with the Bonferroni correction applied to adjust for multiple comparisons. The results revealed a statistically significant difference in sleep scores between the Health Management major and the Clinical Medicine major (*p* = 0.001, adjusted *p* = 0.010). Furthermore, the difference between the Medical Laboratory Science major and the Clinical Medicine major approached, but did not reach the threshold for statistical significance following adjustment (*p* = 0.040, adjusted *p* = 0.401). In contrast, no other pairwise comparisons—such as those between Health Management and Medical Laboratory Science, Health Management and Preventive Medicine, or Medical Laboratory Science and Preventive Medicine—yielded statistically significant differences after adjustment (adjusted *p* > 0.05). These findings indicate that students in the Clinical Medicine major had significantly higher SRSS scores (indicating poorer sleep quality) than those in both the Health Management and Medical Laboratory Science majors. No other significant differences were observed among the remaining majors. See Table 4 for details.

## 4. Discussion

### 4.1. Alarming Prevalence of Poor Sleep Quality Among Medical Students

Sleep quality among medical students presents a significant concern. Our findings reveal that 40.1% of the 551 medical students assessed using the SRSS experienced varying degrees of sleep problems, a rate consistent with the survey results reported by Jeon et al. [23], further underscoring the pervasive nature of poor sleep quality in this population. Recent international studies have confirmed this finding, with Vidović et al. [9] and Khaled et al. [8] reporting that 67.9% and 88.1% of medical students, respectively, experience sleep problems. A systematic review by Seoane et al. [24], which included 29 studies worldwide, found that approximately 39.8% of medical students experience sleep disturbances, which are significantly associated with academic performance. Although different measurement tools were used across these studies, they collectively indicate that medical students are a high-risk group for sleep disorders.

Compared to the national norm, the medical students in this study demonstrated significantly higher scores in subjective sleep quality, insufficient arousal, and post-insomnia responses. This indicates they experienced poorer perceived sleep quality, lower daytime alertness, and more pronounced physical and mental after-effects (such as fatigue and difficulty concentrating) following insomnia. This pattern may be closely associated with the high academic pressures inherent to medical education, including intensive coursework and clinical rotations. This interpretation is supported by recent studies: Gu et al. [25], in a longitudinal diary study of Chinese medical students, further confirmed that sleep quality directly influences next-day positive and negative emotions, suggesting that sleep problems not only affect academic performance but are also closely tied to daily emotional well-being. Similarly, Oberleitner et al. [26] used biometric tracking devices and found that nearly 30% of medical students experienced frequent short sleep (<6 h per night), and that consecutive short sleep was significantly associated with higher levels of stress and depressive symptoms. Conversely, the students scored significantly lower than the norm on early awakening, sleep instability, and nightmare-induced awakenings (*p* < 0.05), suggesting better sleep continuity and a lower frequency of nightmares. This could potentially be attributed to increased sleep drive resulting from chronic fatigue, potentially facilitating a quicker transition into deep sleep stages. Furthermore, structured daily routines, such as fixed clinical rotation schedules, may indirectly contribute to reduced nighttime awakenings.

It is noteworthy that the impact of sleep problems during medical school may extend into the early stages of one’s professional career. A study by Gopika et al. [27] of 150 clinical postgraduates in India found that 62.7% experienced poor sleep quality, and that those with severe stress had an 11.6-fold increased risk of poor sleep, suggesting that the association between sleep and mental health remains significant after graduation. Vidović et al. [9] also found that poor sleep quality was significantly associated with higher levels of depression, anxiety, and stress among medical students, with 42.5% of participants in their study considering seeking professional psychological help. After entering postgraduate training, physicians face additional challenges such as shift work, night duties, high-stakes clinical decision-making, and emotional exhaustion from patient care—all of which may further exacerbate sleep problems. Therefore, paying attention to sleep quality among medical students is of great significance not only for their current academic performance and health, but also for their future career development and patient safety.

### 4.2. Tracing the Sources of Heterogeneity in Sleep Quality Findings Among Medical Students

Our results indicated no significant differences in sleep quality based on gender or academic year among the medical students. This finding, however, contrasts with reports from other scholars. Studies by Malik et al. [18,28,29] concluded that female medical students experienced poorer sleep quality than their male counterparts. This discrepancy could be attributed to several factors specific to female students: psychologically, women often exhibit greater emotional sensitivity, where emotional processing often predominates, leading them to experience a higher prevalence of mental health issues such as depression, anxiety, and other psychological distress compared to males of the same age. Physically, female physiological characteristics, such as pain, anxiety, and irritability associated with dysmenorrhea during menstruation, can also disrupt sleep [23]. Furthermore, research by Zhang Dan et al. [30] demonstrated a decline in sleep quality as students progressed to higher academic years, potentially linked to increasing pressures related to postgraduate entrance examinations and employment. The divergence between these studies and our own can be interpreted through the contextual specificities of our cohort:

Firstly, potent common stressors, such as the intense academic workload inherent to medical education, may supersede and mask more subtle gender-based differences, affecting male and female students equally. Secondly, within the unique geographical and educational context of Hainan, even junior students might encounter distinctive adaptation challenges early on—such as acclimatization to the tropical climate and a densely packed curriculum—thereby mitigating the expected variation across academic years. Additionally, differences in sampling strategies, sample sizes, and measurement instruments across studies are potential contributors to the inconsistent findings. This underscores the necessity of tailoring interventions to the specific local context, rather than indiscriminately applying models developed in other regions.

### 4.3. The Comparative Disadvantage in Sleep Quality Among Clinical Medicine Majors

Students in the Clinical Medicine major demonstrated significantly poorer sleep quality compared to those in the Health Management major. This disparity is likely attributable to the distinctive professional demands of clinical training, including its high-intensity curriculum, practice-related pressures, and potential night shift responsibilities. This finding aligns with the existing research. Clinical medical students consistently report elevated stress levels, which are strongly correlated with sleep disturbances. Their stressors are multifaceted, encompassing heavy academic workloads, the challenges of patient communication during clinical practice, and the psychological burden of dealing with critically ill patients [31].

Compared to other majors, the curriculum for clinical medicine is notably more condensed and academically demanding, leading to the cumulative effect of academic pressure. Furthermore, the heightened educational requirements set by hospitals at various levels compel medical students to extend their years of study. Typically, graduates of clinical medicine bachelor’s programs spend six months to a year preparing for the National Postgraduate Entrance Examination. During this preparation period, they must simultaneously balance clinical rotation responsibilities, including departmental assessments and managing doctor–patient relationships. These competing demands collectively contribute to exacerbating student stress [32,33].

It is worth noting that the survey was conducted in April 2024, during a regular teaching period in the spring semester. Clinical Medicine students typically face intensive coursework and clinical clerkship responsibilities, with some year levels undergoing periodic assessments. In contrast, Health Management students primarily attend theoretical lectures, with less concentrated assessment pressures. A study among medical students in Anhui Province found that students with 45–50 class hours per week had significantly higher PSQI scores than those with lighter academic loads [34], indicating a quantitative association between academic workload and sleep quality. Similarly, a study from Vietnam reported that the prevalence of poor sleep quality among clinical-phase students (51.8%) was significantly higher than that among pre-clinical students (40.0%, *p* = 0.005) [35], consistent with our finding that Clinical Medicine students had poorer sleep quality than their Health Management counterparts.

### 4.4. Limitations

This study has several limitations. First, the cross-sectional design precludes causal inference and can only reveal associations between sleep quality and related factors. Second, the survey was conducted in April 2024, during a regular teaching period, and thus did not capture sleep patterns during high-pressure periods such as examination weeks, which may have led to an underestimation of the peak levels of sleep problems among medical students.

Future research could be extended in the following directions: employing longitudinal designs to track the trajectory of sleep quality among medical students from undergraduate to residency training, thereby helping to elucidate causal relationships and identify critical transition points; incorporating assessments at multiple time points, including examination weeks, to capture dynamic changes in sleep problems; and combining environmental monitoring data with individual lifestyle factors in multicenter, large-sample studies to explore the mechanisms through which tropical climate conditions influence sleep, thereby providing more robust evidence for the development of targeted interventions.

## 5. Conclusions

This study confirms a high detection rate of poor sleep quality among medical students, with Clinical Medicine majors exhibiting significantly poorer sleep quality compared to Health Management majors. Based on these findings, academic institutions and relevant departments should prioritize this public health issue. Particularly in the unique tropical context of Hainan, targeted efforts are needed to optimize the curriculum and clinical rotation schedules for Clinical Medicine students, alongside establishing dedicated psychological support systems. These measures are crucial for effectively alleviating student stress and fundamentally improving their sleep quality and overall health outcomes.

## Figures and Tables

**Table 1:** Demographic Characteristics of the Survey Participants.

Category	Option	Number	Percentage
Gender	Male	197	35.8%
	Female	354	64.2%
Ethnicity	Han	457	82.9%
	Li	50	9.1%
	Other	44	8.0%
Geographical Origin	Urban	269	48.8%
	Rural	282	51.2%
Grade	2023 (Freshman)	102	18.5%
	2022 (Sophomore)	346	62.8%
	2021 (Junior)	27	4.9%
	2020 (Senior)	76	13.8%
Academic Major	Clinical Medicine	139	25.2%
	Preventive Medicine	58	10.5%
	Health Management	285	51.7%
	Medical Laboratory Science	59	10.7%
	Other	10	1.8%
Household Registration	Hainan	243	44.1%
	Other Provinces	308	55.9%
Birth Order	Eldest	256	46.5%
	Second	159	28.9%
	Third	24	4.4%
	Other	13	2.4%
	Only Child	99	18.0%
Monthly Allowance (CNY)	<1000	20	3.6%
	1000–1499	134	24.3%
	1500–1999	162	29.4%
	2000–2499	165	29.9%
	2500–2999	32	5.8%
	>3000	38	6.9%
Grade Point Average (GPA)	<2	11	2.0%
	2–2.49	43	7.8%
	2.5–2.99	98	17.8%
	3–3.49	211	38.3%
	>3.5	188	34.1%
Student Leadership Role	Yes	189	34.3%
	No	362	65.7%
Paternal Education	Junior high & below	323	58.6%
	High School	110	20.0%
	College & above	118	21.4%
Maternal Education	Junior high & below	360	65.3%
	High School	92	16.7%
	College & above	99	18.0%
Family Structure	Nuclear Family	426	77.3%
	Extended Family	43	7.8%
	Single-Parent Family	46	8.3%
	Reconstituted Family	21	3.8%
	Other	15	2.7%

**Table 2:** Comparison of SRSS Scores for Each Factor with the National Norm (x ± s).

Factor	Medical Students (*n* = 551)	National Norm (*n* = 13,273)	*p*
Sleep Insufficiency	2.77 ± 0.89	2.80 ± 0.87	0.4281
Sleep Quality	2.57 ± 0.93	2.33 ± 0.81	0.0000
Insufficient Arousal	2.24 ± 1.11	2.43 ± 1.07	0.0000
Sleep Duration	2.35 ± 0.63	2.30 ± 0.63	0.06801
Difficulty Falling Asleep	2.08 ± 1.07	1.98 ± 0.99	0.0206
Sleep Instability	1.83 ± 0.974	2.00 ± 1.01	0.0001
Early Awakening	1.89 ± 1.06	2.00 ± 1.05	0.0160
Nightmare-induced Awakening	1.66 ± 0.91	2.07 ± 1.11	0.0000
Medication Use	1.22 ± 0.63	1.24 ± 0.64	0.4720
Post-insomnia Response	3.17 ± 1.38	2.96 ± 1.45	0.0008
Total SRSS Score	21.78 ± 5.73	22.14 ± 5.48	0.1315

Unit: points, x ± s.

**Table 3:** Univariate Analysis of Factors Associated with SRSS Scores Among the Medical Student Cohort (*n* = 551).

Item	t	F	H	r	*p*
Gender	−1.59				0.112
Age				−0.001	0.991
Ethnicity		0.729			0.483
Geographical Origin	1.812				0.071
Grade		1.095			0.351
Academic Major			12.791		0.012
Household Registration	−1.186				0.236
Birth Order		0.392			0.814
Monthly Allowance			7.901		0.162
GPA		1.003			0.406
Student Leadership Role	−0.49				0.833
Paternal Education Level			1.758		0.415
Maternal Education Level		0.002			0.998
Family Structure		2.016			0.091

**Table 4:** Pairwise Comparisons of Sleep Scores Across Different Majors.

Pairwise Comparison	Test Statistic	Standard Error	Standardized Test Statistic	*p*-Value	Adjusted *p*-Value
Health Management vs. Medical Laboratory Science	−3.284	22.733	−0.144	0.885	1.000
Health Management vs. Other Majors	−14.276	51.134	−0.279	0.780	1.000
Health Management vs. Preventive Medicine	43.663	22.894	1.907	0.057	0.565
Health Management vs. Clinical Medicine	53.988	16.443	3.283	0.001	0.010
Medical Laboratory Science vs. Other Majors	−10.992	54.352	−0.202	0.840	1.000
Medical Laboratory Science vs. Preventive Medicine	40.379	29.388	1.374	0.169	1.000
Medical Laboratory Science vs. Clinical Medicine	50.704	24.695	2.053	0.040	0.401
Other Majors vs. Preventive Medicine	29.386	54.420	0.540	0.589	1.000
Other Majors vs. Clinical Medicine	39.712	52.036	0.763	0.445	1.000
Preventive Medicine vs. Clinical Medicine	10.326	24.844	0.416	0.678	1.000

Note: The significance level is 0.05. *p*-values have been adjusted using the Bonferroni correction to control the error rate for multiple comparisons.

## Data Availability

The data are not publicly available due to privacy restrictions.
